# Is Motor Milestone Assessment in Infancy Valid and Scaled Equally Across Sex, Birth Weight, and Gestational Age? Findings From the Millennium Cohort Study

**DOI:** 10.3389/fpsyg.2021.781602

**Published:** 2022-01-05

**Authors:** Denise de Almeida Maia, Farid Bardid, Tobias Koch, Paola Okuda, George Ploubidis, Anders Nordahl-Hansen, Michael Eid, Hugo Cogo-Moreira

**Affiliations:** ^1^Department of Psychiatry and Medical Psychology, Federal University of São Paulo, São Paulo, Brazil; ^2^School of Education, University of Strathclyde, Glasgow, United Kingdom; ^3^Department of Psychology, Friedrich-Schiller-Universität Jena, Jena, Germany; ^4^Department of Social Science, Centre for Longitudinal Studies, Institute of Education, University College London, London, United Kingdom; ^5^Department of Education, ICT and Learning, Østfold University College, Halden, Norway; ^6^Department of Education and Psychology, Freie Universität Berlin, Berlin, Germany

**Keywords:** motor development milestones, assessment, infants, confirmatory factor analysis, differential item functioning

## Abstract

Is the assessment of motor milestones valid and scaled equivalently for all infants? It is not only important to understand if the way we use gross and fine motor scores are appropriate for monitoring motor milestones but also to determine if these scores are confounded by specific infant characteristics. Therefore, the aim of the study is to investigate the latent structure underlying motor milestone assessment in infancy and measurement invariance across sex, birth weight, and gestational age. For this study, the birth cohort data from the United Kingdom Millennium Cohort Study (MCS) was used, which includes the assessment of eight motor milestone tasks from the Denver Developmental Screening Test in 9-month-old infants (*N* = 18,531), depicting early motor development of the first children of generation Z. Confirmatory factor analyses showed a better model fit for a two-factor structure (i.e., gross and fine motor development) compared to a one-factor structure (i.e., general motor development), and multiple indicators multiple causes modeling revealed no differential item functioning related to sex, birth weight, and gestational age. The study provides support for the use of gross and fine motor scores when assessing motor milestones in infants—both boys and girls with different birth weights and of varying gestational ages. Further investigation into widely adopted assessment tools is recommended to support the use of valid composite scores in early childhood research and practice.

## Introduction

Motor development plays an important role in children’s health and growth and is crucial for daily life activities; it can be defined as the development of motor skills, which are goal-oriented tasks requiring voluntary movements of one or more body parts (Gallahue et al., 2012). Motor skills emerging before children attain bipedal locomotion are referred to as motor milestones (Burton and Miller, 1998). These rudimentary movement abilities (e.g., sitting, standing, reaching, and grasping) form the foundation for fundamental and specialized motor skills in later childhood onward (Gallahue et al., 2012). Motor milestones are also linked to the social and cognitive development of infants (Burton and Miller, 1998; Collet et al., 2019). Moreover, in view of the childhood obesity pandemic and the focus on preventive approaches, motor skill development from early childhood onward has been recognized as critical to promote lifelong physical activity and health (Stodden et al., 2008; Whitall et al., 2020). It is therefore important that we assess motor milestones adequately in order to support infant and child health and development.

According to the American Academy of Pediatrics and *Bright Futures* guidelines, developmental milestones surveillance should be incorporated in routine health examinations in order to assess and monitor motor skill development among young children (Sices, 2007; Gerber et al., 2010; Mrozek-Budzyn et al., 2014). There are a plethora of motor skill assessment tools used in different contexts including clinical and educational settings (Bardid et al., 2019b). Examples of assessments for infants are the Ages and Stages Questionnaire (ASQ; Squires et al., 1997), Alberta Infant Motor Skills Assessment (AIMS; Piper et al., 1992; Folio and Fewell, 2000), Bayley Scales of Infant Development (BSID; Bayley, 1969, 1993, 2006), Denver Developmental Screening Test (DDST; Frankenburg and Dodds, 1967, 1992), Early Motor Questionnaire (EMQ; Libertus and Landa, 2013), Mullen Scales of Early Learning (MSEL; Mullen, 1995), and Peabody Developmental Motor Scales (PDMS; Folio and Fewell, 2000).

The choice of assessment depends on a range of aspects such as purpose of assessment (Bardid et al., 2019b). There are various purposes for assessing motor skills including evaluation and screening of infants who may be at risk of developmental delay; planning and design of interventions which relies on information from assessments; and monitoring progress as part of child developmental surveillance or to evaluate intervention effectiveness (Burton and Miller, 1998; Johnson et al., 2015) Motor skills can be assessed directly by trained examiners (practitioner or researcher) and/or through parent reports (Bedford et al., 2013). Standardized examiner-administered assessments (e.g., AIMS and PDMS) provide a more accurate estimate of motor skills with less bias and measurement error (Bardid et al., 2019b). In contrast, parent reports (e.g., ASQ and EMQ) are more cost-effective and draw on the primary caregiver’s knowledge (Libertus and Landa, 2013). Parent reports are also useful for measuring large numbers of children [e.g., Lopez Boo et al., 2020; moreover, reports such as the ASQ and EMQ are considered suitable to be implemented in routine health care (Kjølbye et al., 2018)].

Psychometric quality is another key aspect when considering motor skill assessments. There is a body of literature on the psychometric properties of motor skill tests during childhood (for recent literature reviews, see Scheuer et al., 2019; Hulteen et al., 2020). Methodological studies have also been conducted with regard to motor milestone assessment in infancy (see Bedford et al., 2013; Kjølbye et al., 2018). These studies have looked at reliability properties. For instance, internal consistency, inter-rater and test-retest reliability have been established for the PDMS-2, a popular instrument used with children aged 0–6 years (Folio and Fewell, 2000; Connolly et al., 2006; Scheuer et al., 2019). Previous studies have also examined validity properties, although these have generally focused on content validity and criterion validity. Content validity refers to the extent to which an assessment tool adequately reflects the construct(s) it set out to measure (Prinsen et al., 2018). For example, a Brazilian study by Zanella et al. (2021) established content validity for the PDMS-2 through a panel of experts and health professionals. Criterion validity—specifically, concurrent validity—refers to how well an assessment tool correlates with a previously established measure (Prinsen et al., 2018). In their study, Libertus and Landa (2013) reported good concurrent validity of the EMQ with the MSEL and PDMS-2. Similarly, concurrent validity has been reported for the ASQ and AIMS with the BSID (see Lee and Harris, 2005). It should be noted, however, that information on other validity properties such as construct validity and measurement invariance is limited.

Many infant assessments (including those mentioned above) use different test scores reflecting gross and fine motor development, and some use total scores reflecting general motor development. However, there is limited empirical evidence to support the validity of these scales (Burton and Miller, 1998; Haywood and Getchell, 2014). Construct validity—specifically, structural validity—refers to the degree to which the scores of an assessment tool adequately reflect the dimensionality of the construct(s) that is measured (Prinsen et al., 2018). For instance, Zanella et al. (2021) examined the construct validity of the PDMS-2 and provided evidence for a two-factor structure, i.e., gross and fine motor development. In another study, dos Santos Chiquetti and Valentini, 2020 found a one-factor (or unidimensional) structure for the Test of Infant Motor Performance (Campbel, 2012) reflecting general motor development. Notwithstanding, construct validity has not been established for many widely used assessment tools. This poses a major issue for practice and raises the question whether composite scores in motor milestone assessment are valid indicators of motor development. Confirmatory factor analysis (CFA), a structural equation modelling technique, is a widely adopted and useful method to assess the internal structure of motor skills and validate the use of composite scores (Valentini et al., 2018; Okuda et al., 2019; Lopez Boo et al., 2020).

Little is also known on measurement invariance; this indicates whether the scores of an assessment tool are equivalent across groups with different characteristics, which are known to have an influence on these milestones (Tenovuo et al., 1988; Livshits et al., 1993; Flensborg-Madsen and Mortensen, 2017). For instance, previous literature has shown that gestational age and birth weight are related to motor milestones (Pin et al., 2010; da Costa Ribeiro et al., 2017; Flensborg-Madsen and Mortensen, 2017; van Dokkum et al., 2018). Differential item functioning (DIF), another technique commonly used in psychometrics (Steinberg and Thissen, 2006; Millsap, 2012), is adopted to evaluate measurement invariance and explore whether individual characteristics (e.g., sex and birth weight) at a given developmental stage might influence the chances of motor milestone achievement. For example, if differential functioning were detected for sitting, it could suggest that girls, pre-term or low birth weight infants may perform this task more easily than boys, term or normal birth weight infant at the same developmental stage. In other words, some milestone tasks may be more difficult to achieve, depending on individual features such as sex, birth weight, and gestational age. It is important to note that although the direct effect of sex on motor milestones has been reported to be close to zero or very small in terms of magnitude (WHO Multicentre Growth Reference Study Group, 2006a; Hamadani et al., 2013; Flensborg-Madsen and Mortensen, 2017), different authors and studies have adjusted for this covariate in different models (Taanila et al., 2005; Kelly et al., 2006; Murray et al., 2007; Tuovinen et al., 2018). As DIF is evaluated at item level (i.e., probability of performing a task given individual characteristics), it is fundamental to evaluate whether motor milestone achievement is influenced by sex due to its frequent use.

In summary, the first aim of the study was to investigate the latent structure of motor milestones in infants using a range of motor milestone tasks (e.g., sitting, standing, walking, and putting hands together) from the DDST (Frankenburg and Dodds, 1967), a widely used and cost-effective child development scale. As mentioned above, gross and fine motor scores are often used in infant assessment tools including the DDST. In their recent study on initial psychometric properties of the Denver II, Lopez Boo et al. (2020) reported a good fit for the structure with gross and fine motor factors—as proposed by the test developers (Frankenburg and Dodds, 1992). Interestingly, the authors found a better fit for an alternative structure with only one motor factor. In view of current assessment practices, this present study will evaluate both a one-factor structure (i.e., general motor development) and a two-factor structure (i.e., gross and fine motor development) using CFA. The second aim of the study was to evaluate measurement invariance across sex, gestational age, and birth weight. For this, DIF was conducted taking into account that motor milestones are correlated.

## Materials and Methods

### Data

Data were used from the Millennium Cohort Study (MCS), a cohort study that follows the lives of approximately 19,000 children born in United Kingdom at the turn of the century—most recent MCS data were collected in 2018 when the participants were 17–18 years old. For this study, only the first wave of the MCS (MCS1, conducted in 2001–2003) was considered, which provided data on motor developmental milestones. The sample consisted of 18,531 individuals (9,028 girls); mean birth weight was 3.317 kg (SD = 0.591); and mean gestational age was 276 days (SD = 14). More details about the variables birth weight and gestational age are given in Table 1.

**Table 1:** Descriptive statistics.

	Count	%
**Birth weight**		
Low (<2,500 kg)	1,334	7.2
Normal (2,500–4,000 kg)	14,973	80.8
High (>4,000 kg)	2,224	12.0
**Gestational age**		
Very pre-term (<32 weeks)	204	1.1
Pre-term (32–37 weeks)	1,167	6.3
Term (>37 weeks)	17,160	92.6

### Measures

The MCS survey included eight items regarding motor milestones from the DDST (Frankenburg and Dodds, 1967). Although the items are included in the DDST, they are widely used in isolation by clinicians to assess children’s motor development (Gerber et al., 2010) and to screen for potential developmental problems (Johnson et al., 2015). Eight motor milestones of the DDST were used to evaluate gross and fine motor milestones in infants. Four items focused on gross motor skills (sitting without support, standing up alone, walking a few steps, and moving from one place to another) and four focused on fine motor skills (putting hands together, grabbing objects, holding a small object, and passing a toy). All items were categorical and corresponded to a specific milestone with three possible answers (1—Yes, Often; 2—Yes, Sometimes, and 3—No), except for one (move about from one place to another), which was a dichotomous item for gross motor development (1—Yes or 2—No) and may correspond to the milestone roll over (first milestone which allow the infant to move from one place to another). The items were read by the interviewer to the primary caregiver (often, the mother), who responded on a card with a list of possible answers.

### Statistical Analysis

Two CFA models were run to examine the latent structure of motor milestones in infant assessment and to test the validity of motor scores used in assessment practice: (a) a one-factor model, and (b) a correlated two-factor model. In the one-factor model, one latent variable underlying all items was defined as representing general motor development. In the two-factor model, there were two latent variables representing gross and fine motor development. These two factors were allowed to correlate with each other.

To evaluate model fit for CFA, we used the following fit indices with cut-off values as proposed by Schermelleh-Engel et al. (2003): comparative fit index (CFI), root-mean-square error approximation (RMSEA), standardized root-mean-square residual (SRMR). The *χ*^2^ was reported but not considered in the models’ fit evaluation as it would have been inflated due to the large sample size; this test has a very large power to detect even negligible model misfit. Therefore, model evaluation will not be based on this value. A RMSEA value less than or equal to 0.05 indicates a good approximate model fit. The value of *p* of the corresponding test of approximate fit should be less than or equal to 0.05. A CFI value for good fit should be greater than or equal to 0.97; however, values between 0.95 and 0.97 are acceptable. A SRMR value less than 0.05 would indicate a good model fit, a value less than 0.1 an appropriate one. The hypotheses of perfect fit can be tested using a *χ*^2^ test, and the corresponding value of *p* should be less than or equal to 0.05.

To evaluate the potential confounding effect of sex, gestational age, and birth weight, we conducted an invariance test using a multiple indicators multiple causes (MIMIC) model based on the moderately nonlinear factor analysis. This approach, recently developed by Bauer (2017), can be applied to test various hypotheses about DIF, testing all parameters of a model of CFA and considering both discrete and continuous covariates.

The MIMIC model was run using the best factor solution (i.e., one-factor or two-factor solution), which allowed for verification of invariance on any parameter, such as means, intercepts, factor loading, and thresholds (Bauer, 2017). As one of the study aims was to examine the potential influence of covariates (i.e., sex, gestational age, and birth weight) through differential functioning of motor milestones, the parameters of interest were the factor loadings (or *discrimination* or *parameter a* of milestones as per item response theory, IRT) and their thresholds (or *difficulties* or *parameter b* as per IRT).

Mplus version 8.2 (Muthén and Muthén, 2017) was used to run all analyses, including all eight motor milestone items as categorical outcomes.

Due to the multilevel design of the MCS, it was defined a complex type of model, defining country (England, Scotland, Wales, and Northern Ireland) and group (advantaged, disadvantaged, and ethnic minorities) clusters. The distribution of the sample is given in Table 2. This adjustment was implemented *via* the COMPLEX command in Mplus, and details on the estimation of standard errors have been described in previous studies (Asparouhov, 2005, 2006).

**Table 2:** Sample distribution.

	Count	%
**England**		
Advantage	4,707	25.4
Disadvantage	4,688	25.3
Ethnic minorities^*^	2,465	13.3
**Wales**		
Advantage	797	4.3
Disadvantage	1,835	9.9
**Scotland**		
Advantage	1,093	5.9
Disadvantage	1,130	6.1
**Northern Ireland**		
Advantage	686	3.7
Disadvantage	1,130	6.1

* Only in England were there places with at least 30% of ethnic minorities.

To check the model fit for the one-factor and the correlated two-factor solutions where only the categorical items were modeled, the WLSMV (weighted least square with mean and variance adjusted) estimator was used (Asparouhov and Muthén, 2010). For the invariance tests, maximum likelihood estimation with robust standard errors (MLR) estimator was used. Both estimators are robust in terms of dealing with the multilevel data structure (see below); however, they accommodate missing data differently. That is, the WLSMV estimator without covariates, as was used for the one-factor and the correlated two-factor solutions, deals with missing data under pairwise deletion and the MLR estimator deals with missing data *via* full information maximum likelihood. MLR was used for addressing the second aim of this study (i.e., invariance testing) due to the model specification where different constraints were added and covariates were predicting such constraints (factor loadings, item thresholds, and factor variances).

## Results

Descriptive statistics of the covariates gestational age and birth weight are presented in Table 1. Table 2 shows the sample distribution across following categories: ethnic minorities (places with at least 30% of ethnic minorities), disadvantages (poorest 25% places according to the Child Poverty Index for England and Wales) and advantages (others). England is the only country which has places with more than 30% of ethnic minorities. Counts and proportions of all motor milestone items are reported in Table 3. More than 90% of infants aged 8–12 months achieved following motor milestones: sit up without support; grab objects using whole hand; pass a toy back and forth from one hand to another; and move about from one place to another. Seventy to ninety percent was often able to stand up while holding onto something; put hands together; and pick up a small object using forefinger and thumb only. Less than 10% was able to walk a few steps alone.

**Table 3:** Score distribution for the eight motor milestone items from the Denver Developmental Screening Test.

Test item	Category	Count	%
Sit up without support	Often	17,679	95.4
	Sometimes	483	2.6
	Not yet	369	2.0
Stand up while holding onto something such as furniture	Often	13,059	70.5
	Sometimes	2,143	11.6
	Not yet	3,329	18.0
Put hands together	Often	15,739	85.0
	Sometimes	1,687	9.1
	Not yet	1,095	5.9
Grab objects using whole hand	Often	18,380	99.2
	Sometimes	118	0.6
	Not yet	34	0.2
Pick up a small object using forefinger and thumb only	Often	16,492	89.2
	Sometimes	1,248	6.8
	Not yet	741	4.0
Pass a toy back and forth from one hand to another	Often	17,547	94.8
	Sometimes	724	3.9
	Not yet	243	1.3
Walk a few steps on his own	Often	1,066	5.8
	Sometimes	1,373	7.4
	Not yet	16,092	86.8
Move about from one place to another	Yes	17,083	92.2
	No	1,448	7.8

There are 984 missing cases not considered in the analysis (missing in all variables).

**Figure 1:**
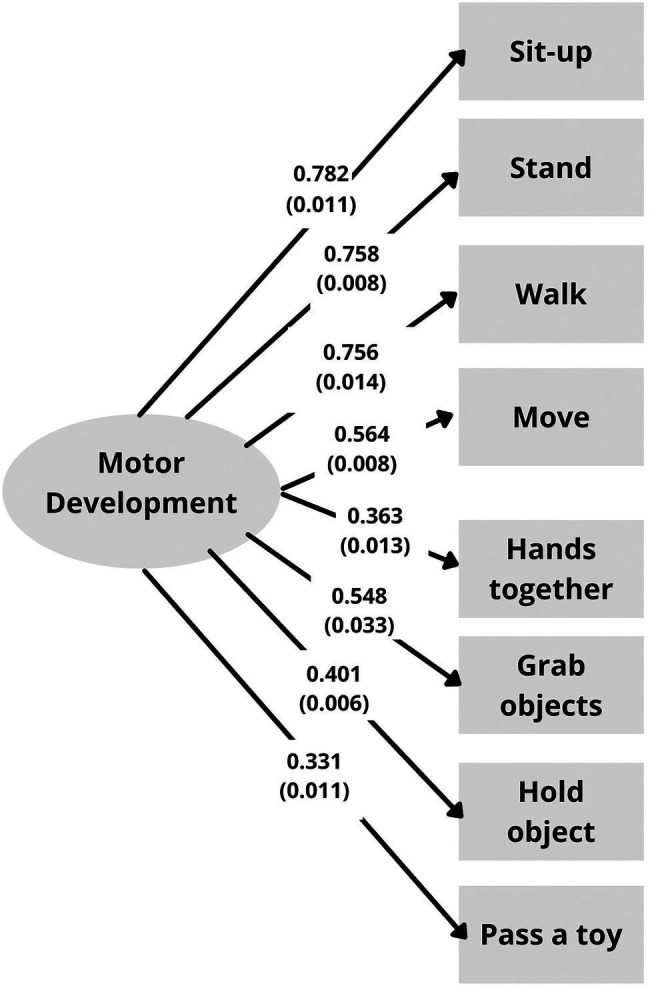
Factor loadings (and standard errors) for the one-factor model.

**Figure 2:**
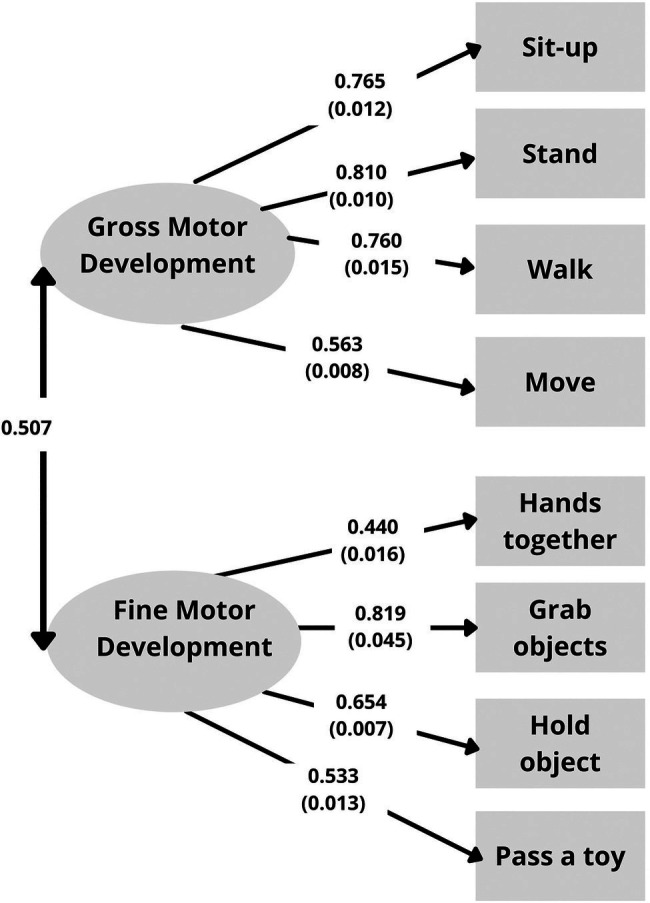
Factor loadings (and standard errors) for the correlated two-factor model.

The factor loadings (and standard errors) of the CFA are shown in Figure 1 (one-factor model) and Figure 2 (correlated two-factor model). The fit indices showed an adequate to good approximate fit for both the one-factor solution, *χ*^2^(20, *N* = 18,531) = 860.443, *p* < 0.001, RMSEA = 0.048, CFI/TLI = 0.894/0.852, SRMR = 0.105, and the correlated two-factor solution, *χ*^2^(19, *N* = 18,531) = 367.640, *p* < 0.001, RMSEA = 0.031, CFI/TLI = 0.956/0.935, SRMR = 0.068. However, the results showed a better model fit for the correlated two-factor model with two latent variables reflecting gross motor development and fine motor development.

The factor loadings and thresholds of the MIMIC model are reported in Table 4. The results showed no indication of DIF for any item with regard to sex, birth weight and gestational age (all *p* > 0.05).

**Table 4:** Results of the multiple indicators multiple causes (MIMIC) model.

Item		Covariate		Factor loadings			Thresholds^*^		
Estimate	SE	*p*	*χ* ^2^	*df*	*p*
Sit up	Birth weight	−0.144	0.156	0.353	0.653	1	0.419
	Gestational age	−0.001	0.006	0.834	1.311	1	0.252
	Sex	−0.115	0.162	0.479	0.001	1	0.979
Stand	Birth weight	−0.040	0.103	0.697	0.021	1	0.884
	Gestational age	0.003	0.003	0.256	0.028	1	0.867
	Sex	−0.073	0.099	0.457	0.555	1	0.456
Hands together	Birth weight	0.059	0.059	0.314	0.006	1	0.941
	Gestational age	−0.001	0.002	0.714	0.114	1	0.735
	Sex	−0.040	0.045	0.367	0.877	1	0.349
Grab objects	Birth weight	0.468	0.292	0.109	0.044	1	0.834
	Gestational age	0.009	0.019	0.643	0.006	1	0.94
	Sex	1.761	1.013	0.082	1.443	1	0.23
Hold object	Birth weight	0.032	0.182	0.859	0.948	1	0.33
	Gestational age	−0.002	0.003	0.509	0.845	1	0.358
	Sex	−0.129	0.078	0.099	0.109	1	0.742
Pass a toy	Birth weight	0.246	0.217	0.257	0.680	1	0.41
	Gestational age	−0.007	0.006	0.220	0.051	1	0.822
	Sex	−0.168	0.292	0.565	0.092	1	0.761
Walk	Birth weight	0.014	0.168	0.934	1.187	1	0.276
	Gestational age	0.002	0.008	0.804	1.009	1	0.315
	Sex	−0.111	0.146	0.447	0.000	1	0.983
Move	Birth weight	−0.098	0.09	0.277	0.906	0.082	0.253
	Gestational age	−0.004	0.003	0.185	0.996	0.003	0.184
	Sex	−0.003	0.082	0.967	0.997	0.081	0.967

* Threshold’s invariances are given *via* Brant Wald Test for Proportional Odds (Chi-square) for those items (all with exception of move about from one place to another) with more than one category of answer and logistic regression odds ratio results for those dichotomous items.

## Discussion

Motor milestones in infancy form the foundation of voluntary movement and motor skills in later life (Burton and Miller, 1998; Haywood and Getchell, 2014). Due to their influence on many facets of child health and development (including social and cognitive), it is important to ensure that motor milestone assessment is valid so as to support researchers and practitioners in their work with children. The purpose of this study was to investigate the latent structure of motor milestones in infants using a set of motor milestone tasks of an existing assessment tool and to examine measurement invariance across relevant infant characteristics (i.e., sex, birth weight, and gestational age).

The first aim of the study was to investigate the construct validity in infant motor milestone assessment. Results of the CFA support the use of eight motor milestone items in measuring gross and fine motor development. A model with two correlated factors showed an appropriate fit; moreover, RMSEA, CFI/TLI and SRMR values showed a better model fit for the two-factor structure compared to the one-factor structure, indicating that gross and fine motor development are two correlated (*r* = 0.507) but sufficiently distinct traits—according to Brown (2015), factors are considered distinct if the correlation between the factors are less than 0.85. As such, these findings support the use of gross and fine motor scores often adopted in assessment tools such as the BSID (Bayley, 1969, 1993, 2006), DDST (Frankenburg and Dodds, 1967, 1992), and the PDMS (Folio and Fewell, 2000). The two-factor solution is in line with previous research. For instance, Zanella et al. (2021) examined the construct validity of the PDMS-2 and reported an adequate fit for a two-factor structure, i.e., gross and fine motor development. Another recent study by Lopez Boo et al. (2020) found a four-factor structure for the complete Denver II including two distinct motor factors (i.e., fine and gross motor development; Frankenburg and Dodds, 1992). Nonetheless, it should be noted that Lopez Boo et al. (2020) found a better fit for a two-factor structure with only one motor factor (i.e., general motor development). Additionally, previous research in older children have found a one-factor structure with one latent trait underlying motor skill assessment (Utesch et al., 2016; Bardid et al., 2019a), supporting the use of total scores in assessment practice. Although the present findings support the use of gross and fine motor scores, it is unclear if total scores could be used in motor milestone assessment. Considering the moderated correlation between both factors and the use of total scores in practice, further investigations are warranted. For instance, future studies could examine to which degree a two-factor structure for infants increases predictive validity in addition to a general factor. This could be done through bi-factor (S-1) modeling (Eid et al., 2017) by taking one factor as a general reference factor and analyzing the incremental validity of a specific factor that is defined by the indicators of that second factor.

The second aim of the study was to investigate measurement invariance across sex, gestational age, and birth weight. Although prior research reported associations between these covariates and infant motor milestones (Peter et al., 1999; Pin et al., 2010; Flensborg-Madsen and Mortensen, 2017; van Dokkum et al., 2018), these studies generally adopted a different methodological approach. For instance, Flensborg-Madsen and Mortensen (2017) investigated a cohort which tracked about 5,000 children, and—through ordinary linear correlations—found low negative correlations of age of milestones attainment with gestational age (−0.19) and birth weight (−0.15). This approach does not disentangling common variance and residual variance. The approach used in this present study is distinct due to its use of structural equation modeling, which takes the latent structure into account, and the influence of sex, birth weight and gestational age thereof. In other words, beyond discussing the impact of these covariates commonly linked to motor milestones, we conducted an item-level analysis of measurement invariance in motor milestones across the covariates, bringing evidence of their “stability” and fairness under different conditions. That is, for two children with the same latent trait of gross and fine motor skills but with different gestational ages (birth weight, or sex), are the probabilities of endorsing item categories differently or even would the items discriminate differently? The present findings show no significant influence of sex, birth weight, and gestational age on factor loadings and thresholds (*p* > 0.05) in motor milestone assessment, in spite of the large sample size (*N* = 18,531). This means that two children with different birth weight (supposing 0.5 kg of difference) but with the same latent trait of gross and fine motor skills would have the same probability of endorsing a give item response; this indicates that assessment of these motor milestones is fair and not influenced by sex, birth weight, and gestational age. These findings are partly supported by previous studies. For instance, the study by Zanella et al. (2021)—examining the validity and reliability of the PDMS-2 in Brazilian young children—found measurement invariance across sex. Although more research is needed, the present findings suggest (Peter et al., 1999; Pin et al., 2010; Flensborg-Madsen and Mortensen, 2017; van Dokkum et al., 2018) that gross and fine motor scores in motor milestone assessment do not only provide a valid measure of gross and fine motor development, but they can also be used to assess and compare infant development across sex, birth weight, and gestational age.

Given our heterogeneous and comprehensive sample—i.e., interval of birth weight (*M* = 3.32 kg, SD = 0.59, min = 0.57, max = 5.80) and gestational age (*M* = 276 days, SD = 14, min = 168, max = 301)—our DIF analysis covered a broad range of individual differences in the covariates. Because the analysis of measurement invariance across different covariates has been seldom done, the results of our study contribute to a deeper understanding of the psychometric quality of motor milestone assessment in the area of pediatric public health, and support the use of gross and motor scores as valid indicators of motor development in infants. It is important to consider gestational age and birth weight for early and targeted intervention, but these covariates do not affect motor milestone assessment. While this study used a large sample and a robust statistical analysis, there are some limitations that need to be considered. First, while parent reports are cost-effective tools suitable for large-scale evaluations, they are arguably less accurate and more susceptible to bias compared to examiner-administered assessments (Bardid et al., 2019b). Further research examining the construct validity and measurement invariance across examiner-administered and parent report measures is needed to support the use of valid test scores in research and practice. Second, the window of time (8–12 months) used to reach the milestones is narrow, which resulted in a low score variance. The World Health Organization (WHO Multicentre Growth Reference Study Group, 2006b) indicates there is much variability in development and infants may be able to achieve independent standing and walking between 7 and 18 months. Therefore, future studies should consider a wider age range. Third, this study only included a relatively small item-set from the DDST (with four gross motor items and four fine motor items), which might restrict the generalizability of the present results. As such, future validity studies should evaluate assessments with larger item-sets. For instance, the EMQ (Libertus and Landa, 2013) consists of 128 items including 49 for gross motor skills and 48 for fine motor skills (Squires et al., 1997; Libertus and Landa, 2013).

It is clear that we should not only assess and monitor motor milestones (Gallahue et al., 2012), but also adopt valid scores for appropriate interpretation. Monitoring motor skill development is critical to support positive developmental trajectories of health. This is particularly important due to changes in lifestyle patterns. More time is now spent in sedentary behavior and less time is spent being physically active (Whitall et al., 2020). Recent studies have examined screen time influences motor skills (Hardy et al., 2018; Webster et al., 2019). In a survey with 1,000 parents in the United States, Zimmerman et al. (2007) found that 90% of children by 24 months of age watch TV, DVD, and videos regularly and that the median age of when children start to watch screens was 9 months old. Furthermore, in view of the increasing levels of childhood obesity in the last decades, researchers have found that competence in fundamental motor skills (e.g., running, jumping, and throwing)—which build on motor milestones—is associated with weight status and should therefore be considered in physical activity promotion and obesity prevention (Stodden et al., 2008; Whitall et al., 2020).

In summary, the current study showed insights into the construct validity of motor milestones in infancy. The study provided evidence for a correlated two-factor structure underlying motor milestone assessment and reflecting gross and fine motor development. These findings support the use of gross and fine motor scores in infant assessment practice, helping health professionals in early detection of delay in motor milestone achievement and provision of targeted support for child development and health. Additionally, the results showed no significant influence of sex, birth weight, and gestational age in motor milestone assessment, indicating that gross and fine motor scores can be used for both boys and girls with different birth weights and gestational age, although these variables should be considered in intervention design and delivery. Further methodological research is needed to examine motor skill assessment in infants and to validate the use of composite scores in widely used motor milestone instruments.

## Data Availability Statement

The datasets presented in this study can be found in online repositories. The names of the repository/repositories and accession number(s) can be found at: http://www.ukdataservice.ac.uk/.

## Ethics Statement

The Millennium Cohort Study was reviewed and approved by the National Health Service Research Ethics Committee in the United Kingdom. Written informed consent to participate in the study was provided by the participants’ legal guardian/next of kin.

## Author Contributions

DA, PO, and HC-M prepared the manuscript. DA, PO, and FB reviewed the literature. DA and HC-M carried out the analyses. TK, ME, and HC-M conducted the analyses and its theoretical aspects in the discussion. FB, GP, and AN-H contributed with the revision of the paper and theoretical aspects in the discussion. All authors contributed to the article and approved the submitted version.

## Funding

This study was financed in part by the Coordenação de Aperfeiçoamento de Pessoal de Nível Superior – Brasil (CAPES) – Finance Code 001. HC-M would like to express his gratitude for the Senior Researcher CAPES-Alexander von Humboldt Post-Doc Fellowship (Process number 88881.145593/2017-01).

## Conflict of Interest

The authors declare that the research was conducted in the absence of any commercial or financial relationships that could be construed as a potential conflict of interest.

## Publisher’s Note

All claims expressed in this article are solely those of the authors and do not necessarily represent those of their affiliated organizations, or those of the publisher, the editors and the reviewers. Any product that may be evaluated in this article, or claim that may be made by its manufacturer, is not guaranteed or endorsed by the publisher.
